# State of Innovation in Alginate-Based Materials

**DOI:** 10.3390/md21060353

**Published:** 2023-06-08

**Authors:** Katarzyna Adamiak, Alina Sionkowska

**Affiliations:** 1 Department of Biomaterials and Cosmetic Chemistry, Faculty of Chemistry, Nicolaus Copernicus University in Torun, Gagarin 7 Street, 87-100 Torun, Poland; alinas@umk.pl; 2 WellU sp.z.o.o., Wielkopolska 280, 81-531 Gdynia, Poland; 3 Faculty of Health Sciences, Calisia University, Nowy Świat 4, 62-800 Kalisz, Poland

**Keywords:** alginate, biomaterials, natural polymer

## Abstract

This review article presents past and current alginate-based materials in each application, showing the widest range of alginate’s usage and development in the past and in recent years. The first segment emphasizes the unique characteristics of alginates and their origin. The second segment sets alginates according to their application based on their features and limitations. Alginate is a polysaccharide and generally occurs as water-soluble sodium alginate. It constitutes hydrophilic and anionic polysaccharides originally extracted from natural brown algae and bacteria. Due to its promising properties, such as gelling, moisture retention, and film-forming, it can be used in environmental protection, cosmetics, medicine, tissue engineering, and the food industry. The comparison of publications with alginate-based products in the field of environmental protection, medicine, food, and cosmetics in scientific articles showed that the greatest number was assigned to the environmental field (30,767) and medicine (24,279), whereas fewer publications were available in cosmetic (5692) and food industries (24,334). Data are provided from the Google Scholar database (including abstract, title, and keywords), accessed in May 2023. In this review, various materials based on alginate are described, showing detailed information on modified composites and their possible usage. Alginate’s application in water remediation and its significant value are highlighted. In this study, existing knowledge is compared, and this paper concludes with its future prospects.

## 1. Introduction

**Figure 1:**
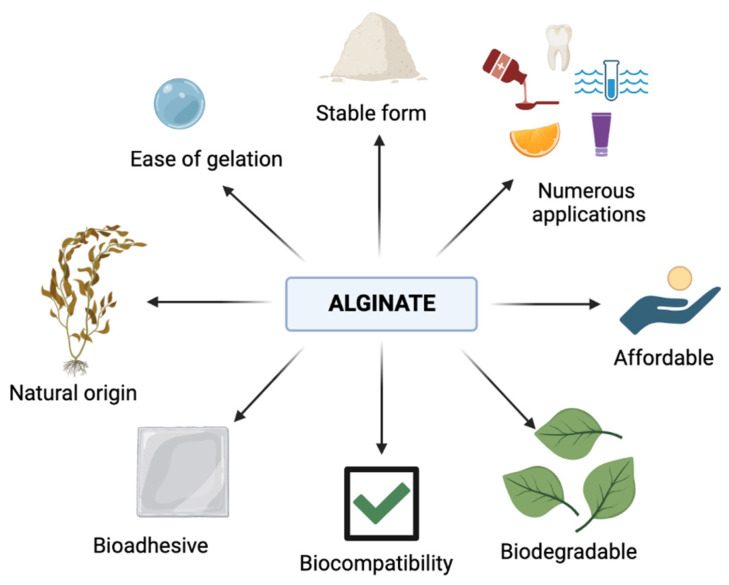
Schematic presentation of alginate properties (graphic was prepared with program BioRender.com, accessed on 18 April 2023).

Alginate constitutes a hydrophilic and anionic polysaccharide originally extracted from natural brown algae (kelp) and bacteria [1,2]. The main species of brown algae as the source of alginates represent *Ascophyllum nodosum*, *Durvillea antarctica*, *Laminaria digitata*, *Laminaria hyperborea*, *Macrocystis pyrifera*, *Ecklonia maxima*, *Laminaria japonica*, *Sargassumspp*, and *Lessonia nigrescens* [3,4,5]. Enzymes such as alginate lyases can degrade these polymers into monosaccharides and oligosaccharides, which leads to major applications [6]. A wide variety of functional features, such as biocompatibility, ease of gelation, and bioadhesive and biodegradable properties, are the main reason why alginate has many applications (see Figure 1).

Alginate materials can be found in the medical, cosmetic, and engineering industries [7,8].

The gel-forming and ion-exchange capability of alginates make them useful in wound-regeneration materials. In recent years, the improvement in these materials enhanced their antimicrobial, absorption, and gel-forming properties. The development of alginate fibers enabled the discovery of new functional biomaterials [9]. Alginate generally occurs as water-soluble Na alginate and is transformed into Ca-alginate beads. These forms are used as adsorbents that remove heavy metal ions. Chelating and mucoadhesive properties are other reasons to use this material in drug delivery systems in the pharmacological field. Moreover, due to its degradation ability and recycling, alginate is considered an environmentally friendly polymer. The physicochemical properties of alginates, such as sol–gel transition, pH responsivity, and thermostability, make alginate materials a fast-developing field. These properties can be modified in the presence of surfactants, crosslinkers, and their concentrations, as well as stirring time manipulations. Using processing parameters and different techniques, it is possible to obtain nanoparticles with a desirable encapsulation efficiency, size, drug release profile, and zeta potential [10,11,12,13].

This review will provide an extensive survey of alginate’s structure, chemical properties, and applications and suggests new perspectives for future studies with these polymers.

## 2. Chemical Structure and Properties of Alginate

**Figure 2:**
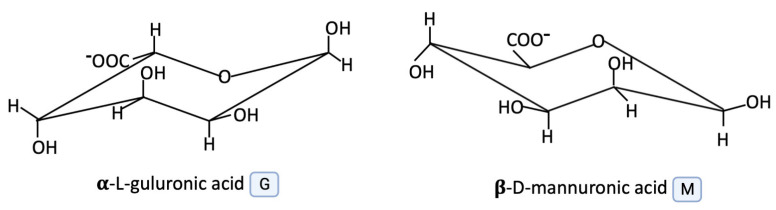
The structure of α-L-guluronic acid and ß-_D_-mannuronic acid (graphic was prepared with program BioRender.com, accessed on 18 April 2023).

Alginate is an unbranched polysaccharide copolymer built from L-guluronic and _D_-mannuronate blocks combined together with 1,4-glycosidic linkages [14]. The structures of α-L-guluronic acid and ß-_D_-mannuronic acid are shown in Figure 2.

**Figure 3:**
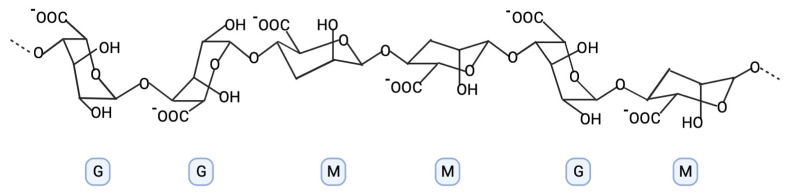
The structure of alginate with the MG and GM residues (graphic was prepared with program BioRender.com, accessed on 18 April 2023).

The _D_-mannuronate component is the main fraction in the precipitation process with Ca salts. The content ratio of mannuronate to guluronate can vary and depends on the source of alginates. Alginate linear copolymers are built with blocks of (1,4)-linked α-L-guluronate (G) and ß-_D_-mannuronate (M), as one can see in Figure 3. Only the G-block structure is involved in the intermolecular covalent crosslinking binding with divalent cations and leads to the hydrogel-forming process. The length of the G-block, M/G ratio, and molecular mass are important agents with an influence on the physical properties of alginates [15,16,17,18].

The molecular mass of alginates varies depending on their origin, whereas bacteria-derived alginates are distinguished by high molecular masses and a high degree of polymerization [19,20]. The process of gelation can be induced by the presence of divalent cations, for example, Ca^2+^, Ba^2+^, or Mg^2+^, which initiates alginate aggregation and forms a physical gel [21]. One of the main parameters that influence the hydrogelling ability of alginate is pH responsivity; material shrinks in at a low pH, whereas it swells and releases the medications from its carriers at higher pH values as a promising drug medium material for oral delivery. The structure of the gel is determined by ion type, sequence, composition, and guluronic residue content [15]. Alginate is supplied in various forms on the market: powder, powder with an activator, and paste. The most common form of alginate is powder, which can be mixed with water. The paste is available in two states that vary in viscosity: tray and syringe viscosity. When comparing the state of the paste to the powder state, the paste requires a shorter gelation time, and studies have shown that the paste form better meets the requirements of the dentistry industry than the powder form [22].

The mechanism of alginates in a natural state can interact with cationic polyelectrolytes and proteoglycans via a pH-dependent anionic nature, whereas in delivery systems for cationic medications, electrostatic interactions are used [7,8].

## 3. Alginate Extraction and Origin

**Figure 4:**
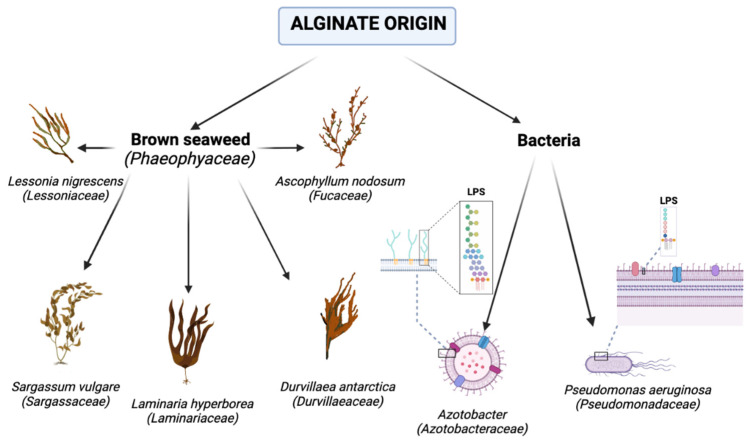
Alginate origin (graphic was prepared with program BioRender.com, accessed on 18 April 2023).

Nowadays, alginates used commercially are extracted from various species of brown seaweed, as can be seen in Figure 4 [23].

**Figure 5:**
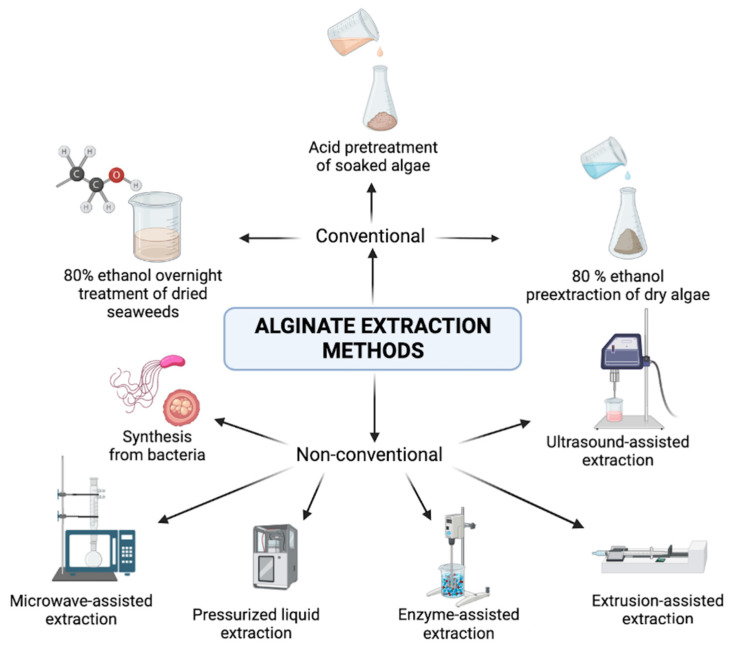
Alginate extraction methods (graphic was prepared with program BioRender.com, accessed on 18 April 2023).

Increasing CO_2_ levels caused by climate change have become a threat to seaweed fields. The alternative methods of laboratory cultivation or obtaining a specific ratio of (1,4)-linked α-L-guluronate (G) and ß-_D_-mannuronate (M) to suit the proper expectations are time- and money-consuming. Therefore, there is an increasing need for customizable sources of alginates. Alginate extraction methods can be divided into conventional and non-conventional, as Figure 5 shows.

Species of *Azotobacter* and *Pseudomonas* also constitute sources of alginate [24,25,26]. Gram-negative bacteria extracted from water, soil, or other natural or laboratory environments, as an opportunistic pathogen, can be a reason for severe infections of urinary, respiratory, or cystic fibrosis infections [27,28]. The genome of *P. aeruginosa* contains many secreted virulence factors, such as exoenzymes, exotoxins, proteases, and lipases [29]. In the outer membrane, the cell of *P. aeruginosa* includes virulence factors such as lipopolysaccharide (LPS) [30]. The main feature of this bacteria is the capability to produce biofilms rich in alginates [31,32,33,34,35]. In *Pseudomonas aeruginosa*, alginate is polymerized and secreted by a specific unit that spans the outer and inner membranes of the bacteria [36,37]. The particular protein in this unit is expressed by a biosynthetic operon with 12 coding sequences [38]. These genes are observed in bacterial species that produce alginate. According to *Pseudomosas aeruginosa*, the expression of this operon is controlled and regulated by AlgU, which is the sigma factor expressed from the alginate regulatory operon [39,40]. Positive alginate biosynthesis regulators act via the degradation of the AlgU regulator to release AlgU, resulting in the expression of the biosynthetic operon and, finally, alginate production [41,42,43]. The biotechnology method of producing alginates from bacteria is complicated due to potential pathogenicity and diversified content in various strains. Other research has shown that the overexpression of the alginate biosynthesis activator MucE is made possible by activating the protease AlgW, which degrades MucA, thus activating AlgU [44]. Alternative methods are based on deleting five virulence factor genes from the chromosome of a type of *P. aeruginosa* strain, which leads to the attainment of a non-pathogenic strain: PGN5. The PGN5 strain can produce high amounts of alginate. Biotechnology methods can make alginates with desired physical properties, improving their features and expanding their application [45].

## 4. Alginate Applications

### 4.1. In Cosmetics

Novel research published by Sayin et al. [46] has shown that alginate extracted from *Sargassum vulgare* can be used as a preservative agent in cosmetics. As the study showed, alginate indicates a higher antimicrobial effect than commercially used herbal preservative 705, which contains glyceryl caprylate and glyceryl undecylenate. The results of the research have shown that alginate from *Sargassum vulgare* needed less time to initialize antimicrobial activity on microorganisms such as *Staphylococcus aureus*, *Pseudomonas aeruginosa*, *Candida albicans*, *Aspergillus brasiliensis*, and *Escherichia coli.* Antimicrobial tests were performed according to the ISO 11930 standard [46]. The literature provides information about the most proper and the best-quality sources of alginates in cosmetic applications, such as *Laminaria hyperborea*, *Laminaria digitata*, and *Laminaria japonica*, whereas the most common source of marine polysaccharides is brown seaweed in the *Phaeophyceae* family, *Macrocystis pyrifera* [47,48,49]. In cosmetics, alginates are used for the encapsulation of oils, which mask their specific undesirable effects and help to maintain their stability. The medium is classified in addition to its structure, the number of cores, and sizes. The encapsulation techniques, where the encapsulating material is alginate, use internal, external, or inverse-gelation mechanisms. Alginates are widely commercially used because of their gelling capacity and biocompatibility [50]. Recent research has shown that sodium alginate can be used with tetradecylallydimethylammonium bromide as shells based on complex coacervation–emulsion polymerization to create apple aroma microcapsules with a potential application in cosmetics [51].

Oils for cosmetic applications such as argan oil, berry oil, caprylic/capric triglyceride, apricot kernel oil, raspberry oil, ginko biloba oil, moringa, *Pistacia Lentiscus* oil, rose hip oil, camellia oil, black wheat oil, and jojoba and soybean oil can be encapsulated by alginate to protect active substances and to obtain a controlled release in skincare and haircare products [52,53].

Novel research has shown that cosmetic patches that enhance the moisture, adhesiveness, and delivery of active substances to the skin are developed using spirulina extract-impregnated alginate nanofiber supported by polycaprolactone nanofiber cover. The tests showed that none of the three components detected cytotoxicity in a human keratinocyte-cell-based examination. Furthermore, the patch caused an increase in moisture and adhesiveness to the skin [54].

The application of alginates is wide. Alginate helps to maintain proper skin or hair moisture in creams, emulsions, hair gels, and face masks, which means it can work as a humectant in cosmetic formulas. It also protects the cosmetic product from stratification of phases in emulsions. It is often used as a gelling agent in acidic pH and consistency regulators.

### 4.2. In Food

The United States Food and Drug Administration (FDA) institution has approved alginate for a variety of applications, such as food, medicine, and supplements [55,56,57,58,59]. In the food industry, alginate is used as a stabilizer as well as an emulsifying and thickening agent. Because of its polysaccharide indigestible structure, alginate can be a source of dietary fiber as well, which helps digestion regulation and works as a prophylactic measure in gastrointestinal and cardiovascular diseases [60,61]. Recently published articles have shown that alginate-based microgels with polymeric and colloidal fillers can enhance probiotic stability during food storage. *Lactobacillus casei* encapsulated by calcium alginate microgels with polymeric or colloidal fillers can increase probiotic viability [62].

Nowadays, the food industry is focused on functional food. Research has shown that cocoa extract can be encapsulated using alginate. The extrusion method can induce either internal or external gelation. Alginate/cocoa beads with calcium prepared via the internal gelation method represented a more homogenous and compact structure, whereas the beads obtained by the external gelation method were harder because of the more rigid shell which formed due to the migration of calcium to the exterior of the beads [63]. The main characteristics of alginates that make them thickeners, stabilizers, and restructuring agents in the food industry are their perfect gelling abilities at low temperatures and thermostability. They can also be used as a carrier for protective coatings for fruits and vegetables [64]. Research has shown that single alginate films exhibit more appropriate features for food packaging applications than single-pectin or composite films [65].

Water solutions of alginate indicate higher viscosities and an exceptional sheer thinning effect compared to other commercially used thickeners. The applied sheer thinning effect while processing can lower the viscosity of the food mixture [66]. Alginate’s thickening abilities are well used in marmalades, jams, and fruit sauces, as alginate–pectin interactions provide heat-reversible effects and higher viscosity. Other applications of alginates in the food industry are thickener agents for desserts, savory sauces, and mayonnaise. The hydrophilic nature of alginates helps to improve the retention of moisture and food texture and provides better organoleptic features to promote customer acceptance [67]. According to the perfect gelling abilities in high and low temperatures and at a low pH, alginates can be used in food processing, for example, in bakery creams in which alginates reduce the separation of the liquid and solid components. In ice cream, alginate is used with other hydrocolloids to control the viscosity of the product, maintain temperature resistance, and reduce shrinkage and ice crystal formation [68,69]. The proper tensile strength and flexibility of alginates make them suitable as an ingredient for food coatings. Once the surface of the food touches the water solution, alginate becomes an edible coating, protecting the product. The film-forming ability of alginates can be a potential answer to replacing non-recyclable food packaging. Alginate and gellan-based coatings are used to prolong the shelf life of fresh-cut Fuji apples packed in trays. The shelf life for control apple slices was four days, whereas using the alginate coatings shelf life for Fuji apples prolonged the shelf life to two weeks [70]. In addition, alginate gels are safe to use and provide better stability for the product and prolong its shelf life [71,72]. Alginate encapsulation techniques, such as spray drying, spray cooling, and spray freezing microfluidization, can provide acidulants, fats, and flavors, which are widely used in the food industry for food processing, improving the functionality and acceptability of the product.

### 4.3. In Medicine

Alginates are commonly used in medicine, tissue engineering, drug delivery, and as a drug component in medications that prevent gastric reflux because of their non-antigenicity, biocompatibility, biodegradability, and chelating ability [7,8]. Furthermore, alginate encapsulation techniques are used in drug delivery applications [73].

The delivery systems for cationic drugs can be received by electrostatic interactions because of the pH-dependent anionic alginate structure and its response to cationic polyelectrolytes and proteoglycans’ capability. Scaffolds are one of the methods of delivering drugs, therapeutical cells, and growth factors into their proper place in the organism and play an essential role in protecting incorporated substances or cells from the biological environment. The scaffold’s time of degradation can be regulated with a proper pH as well as mechanical or swelling properties. Kun Ma et al. [74] have shown that alginate in fibrin/alginate blended hydrogels used in cartilage tissue engineering, especially for the support of bone-marrow-derived mesenchymal stem cells, enhance the gel biostability and support collagen II and glycosoaminoglycan production and chondrogenic gene expression. Moreover, alginate scaffolds seeded with cardiomyocytes can prevent deterioration in cardiac function after myocardial infarction in rats [75]. Chitosan–alginate hybrid scaffolds are used in bone tissue engineering. Li et al. [76] showed that the chitosan–alginate scaffold could be prepared from solutions of physiological pH. This approach provided a favorable environment for incorporating proteins with less risk of denaturation. Moreover, alginate scaffolds can deliver heparin-binding angiogenic factors or a combination of growth factors such as VEGF, PDGF-BB, and TGF-B1 in various tissue-regeneration processes [77].

The degradation time and mechanical properties can also be successfully regulated by the molecular weight of alginate—the higher the molecular weight, the slower the degradation rate, which influences the mechanical properties of biomaterial via structural changes [78,79]. As research has shown, the potential of alginate in regenerative medicine is very promising; thus, the demand for alginate-based biomaterials is still rising [80,81,82]. Tested alginate-based materials indicate possible vascularization, low inflammatory response, degradation, and protection of wounds from bacterial infection. Depending on the character of a wound, the wound dressing should maintain accurate properties; for example, in some cases, it should provide a moist occlusion dressing to help the skin heal in a shorter time. There are many kinds of alginate-based wound dressings, such as hydrogel, sponges, and electrospun mats [83,84,85]. The antiseptic capability and decreased tissue regeneration time provided by better cosmetic repair of the wounds, as well as easy gel-forming ability, are the significant advantages of alginates in this field [86]. Moreover, alginate-based materials in various forms, such as microspheres or injectable hydrogels, are used in cartilage regeneration and broadly defined tissue engineering [87,88,89,90,91,92,93,94]. Furthermore, reconstructive bone therapy that utilizes adequate scaffolds with growth factors or cells may improve the process of tissue regeneration [95,96,97,98,99,100]. Several studies have shown that alginate is successfully used in scaffolds to improve healing [100,101,102,103,104,105,106,107]. Alginate materials are commonly used in drug delivery systems as colloidal particles, hydrogels, porous scaffolds, and polyelectrolyte complexes, as well as microspheres [108,109,110,111,112].

### 4.4. In Dentistry

**Figure 6:**
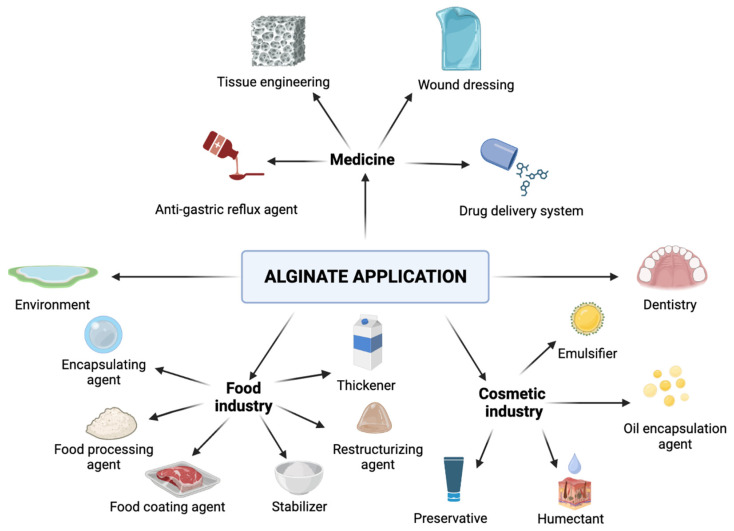
Alginate application (graphic was prepared with program BioRender.com, accessed on 18 April 2023).

Alginate materials are well used in dentistry as adhesives and impression materials that can be painted or sprayed on the tissue. Before use, alginates are provided in powder form, which can be mixed with water. It is crucial to use demineralized water because metal cations can affect the accuracy and setting of the alginate. The obtained mixture should be creamy, without air in it. After this step, the mixture can be placed in the patient’s arch, and then alginate can flow into the cavities and record details. After a short time, the dentition fingerprint is taken by a quick snap, washed, disinfected, and stored in a waterproof package. However, the impression materials should not be stored for too long because of possible dental inaccuracies in time [113,114,115,116,117,118,119]. The double impression technique uses thermo-plastic pastes that are composed of fluid paste and basic paste which, before usage, should be activated with proper catalysts. The elastomers are more stable than alginates but require a good and quick technique of application [120]. Digital fingerprinting techniques are promising alternatives to the standard techniques in order to obtain the most precise fingerprint and durable material [121]. The summary of alginate applications is shown in Figure 6.

### 4.5. In Environmental Protection

The industrialized environment produces waste containing heavy metals, which enter the trophic chain and are transferred from soil, water, plants, and animals to humans [122].

Heavy metals accumulate in the organism and can cause changes in protein synthesis, disturbances in ATP production, and, in the end, carcinogenic changes [123]. To prevent heavy metal ions from transferring to the environment, a study showed that alginate-based materials could be low-cost and effective adsorbents for heavy metals such as Pb (II), Cu (II), and Cd (II) from aqueous solutions as they contain carboxyl and hydroxyl groups [124].

Although sodium alginate has some restrictions, such as thermal degradation and poor stability, the modifications and alginate composites help to provide the expected characteristics for each application [125]. Heavy metal ion adsorption is based on physicochemical interactions between metal ions and the functional groups on the surface of the adsorbent, including electrostatic interactions, chelation, complexation, reduction, and ion exchange. In alginate-based adsorbents, hydroxyl groups are responsible for these interactions [126]. The heavy metal retention capacity is dependent on the pH value of the solutions comprising metals, which have an influence on the surface charge of absorbates and adsorbents. Low pH values cause the decreased adsorption of metals such as Cd^2+^, Cu^2+^, Zn^2+^, Co^2+^, and Ni^2+^, whereas due to deprotonation, anionic metals such as Au(CN)^2−^, AuCl^4−^, PtCl_3_^4−^, CrO_2_^4−^, and SeO_2_^4−^ indicate increased adsorption [127].

Next to heavy metal ions, environmental pollution is also caused by synthetic dyes entering water systems. The pharmaceutical, paper, textile, and food industries use synthetic dyes in tons, but due to improper processing, it is considered a major environmental problem as it is highly toxic [128]. Cationic dyes can interact with negatively charged cell membranes and can easily transfer into the cell cytoplasm [129]. Anionic dyes are less toxic than cationic dyes. Crystal violet dye is a common basic dye used in the textile, veterinary, and food industries [130]. Crystal violet dye is regarded as a mutagen and mitotic poison [131,132]. Magnetite alginate beads constitute a promising, efficient, and low-cost method for the removal of crystal violet dye from aqueous solutions. The limitation of using alginate individually is that the sorbent without magnetite showed a low regeneration capacity and could be used one time with proper efficiency. Magnetite alginate can be effectively used up to four times using 0.01 M HCl, which means the sorbent is a promising alternative for removing dye from industrial wastewater [133]. Magnetic alginate beads also have potential to be used as a sorbent for lanthanum (III) from the aqueous solutions regenerated by 0.1 M CaCl_2_ with optimal efficiency [134]. Lanthanum is used in catalysts, batteries, and the ceramics industry [135,136] due to its unique physicochemical properties. The occurrence of this chemical element is rare. It can be found in monazite, cerite, bastnasite, and allanite earth [137].

Nevertheless, high concentrations of lanthanum entering the environment, especially water, is considered toxic waste. Therefore, it is necessary to develop adsorbents to efficiently remove lanthanum from water to protect the environment and recover a rare chemical element.

## 5. Comparison of Existing Knowledge in the Field of Alginate-Based Materials

The structure of alginates has a significant number of free hydroxyl and carboxyl groups located along the polymer chain; thus, in contrast to neutral polysaccharides, they have two types of functional groups that can undergo modification. The modification process can lead to a change in the properties of a mentioned compound. There are several methods that are commonly used regarding hydroxyl groups, such as alginate-reductive amination, copolymerization, coupling cyclodextrin units, sulfation, and oxidation. Other possibilities, including modification in carboxyl groups, use amidation, esterification, and Ugi reaction [138,139].

Novel techniques use cryotropic gelation with non-deep freezing of the solution containing proper precursors, its storage while frozen and thawing, and obtaining cryogels. Cryogels have a high amount of interconnected macropores as well as gigapores, where sizes are up to 100 µm. These pores are created by growing crystals in the solvent [140]. The method of cryogelation can be divided into cation-free or ionotropic cryogelation. In the extraction process, alginate is surrounded by a non-solvent that can strengthen the polymer structure. However, supercritical drying to obtain macroporous alginate has not been conducted yet [141,142,143,144,145,146]. In the 1960s, several laboratories used non-solvent-induced Phase Separation by Loeb and Sourirajan [147], in which a polymer solution separates into polymer-rich and polymer-lean phases as the solubility of the macromolecules decreases because of the addition of non-solvent. This technique is used in polymer technology to diversify a set of synthetic and non-synthetic polymers [148]. Recently, the non-solvent features of solvents were used to improve the strength of the alginate material [149,150,151]. Carbon dioxide-induced gelation is a method that obtains even giant monoliths [152]. It began in 1990 and was performed by Draget et al. [153]. The modification of this method was recently developed by Gurikov et al. [154,155]. Another method is based on the generation of protons via water electrolysis in a water solution of sodium alginate. The crosslinking process of alginate can be delivered using Ca^2+^ or Fe^3+^ cations [156]. There were also approaches for electrospinning alginate in concentrated solutions of calcium, magnesium, or barium salts [157]. The literature shows that alginate is biocompatible, non-toxic, and biodegradable when administered orally [158]. However, there is a disagreement when it comes to intravenous alginate administration. Tests have shown that there was a foreign body and fibrosis reaction while using it intravenously [159,160], while different reports have showed a slight or no response at all after using alginate implants [161]. In most cases, alginate after free-flow electrophoresis purification does not cause foreign body reactions [162]. The immunogenic response to alginate by intravenous administration may be caused by toxic contaminants from commercial alginates [163].

Polymers with charge density can be mucoadhesive agents [164,165,166]. Polyanionic polymers are reportedly more effective as bioadhesives than polycation polymers or nonionic polymers [164]. Therefore, alginate with carboxylic groups is a promising mucoadhesive agent. Compared to chitosan, polylactic acid, carboxymethyl cellulose alginate has the highest mucoadhesive strength [165]. Although alginate is successfully used in many applications in food, cosmetics, dentistry, and medicine, the form of calcium alginate has its limitations. However, the method used to obtain the form of calcium alginate is simple; the loss of the compound during this process by bonding the created pores is significant [167,168]. Therefore, there are many reports in the literature about the crosslinking process of alginate. Sodium alginate [169,170,171] or sodium alginate with gelatin or ovalbumin [172] was crosslinked with aldehydes [169]. Chan et al. in 2002 [169] used pentane diol with two aldehyde groups to create crosslinkage between two alginate molecules by the formation of two hydroxyl groups via pentanedial. Other methods discussed in this chapter overcome the limitations caused by a relatively large pore size and instability in higher pH environments [163]. Developing new systems for nanotechnology using natural biopolymers is an open issue [173]. We can expect that alginate will play an important role in this field as well.

**Figure 7:**
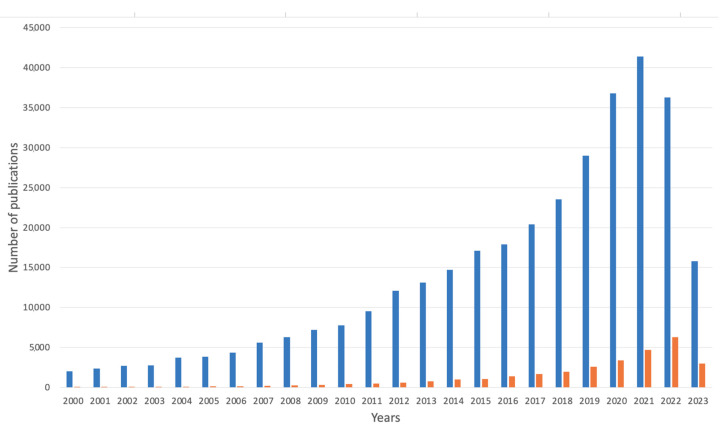
Number of publications over the years on alginate-based materials in environmental field (search for words “alginate” and “environment” together) and products based on alginate in this field (search for the phrase “alginate-based” and “environment”). Data are provided from Google Scholar database (in both matters including abstract, title, and keywords); accessed on 18 April 2023.

Currently, alginate-based materials are gaining more recognition. The scientific literature in the year 2000 had a total of 2020 results for the words “alginate” and “environment” together and 45 results for “alginate-based, environment” for products with an alginate base in environmental remediation (see Figure 7). In the year 2022, the number of publications in the first example raised to 36,300, while in 2023, the results numbered 15,800, whereas the number of publications for products based on alginate was 6300 for the year 2022 and 3000 for the year 2023. The results were obtained using the Google Scholar database in both cases, including the abstract, title, and keywords. The total list of publications was 367,047 (date of access: May 2023).

**Figure 8:**
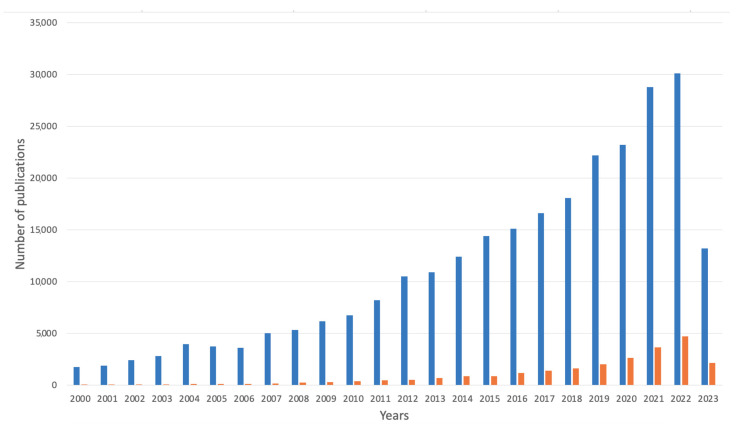
Number of publications over the years on alginate-based materials in medicine field (search for words “alginate” and “medicine” together) and products based on alginate in this field (search for the phrase “alginate-based” and “medicine”). Data are provided from Google Scholar database (in both matters including abstract, title, and keywords); accessed on 18 April 2023.

Alginate-based materials in medicine in the years 2000–2023 showed a comparatively large increase. The total amount of publications in the Google Scholar database when searching for the phrases “alginate” and “medicine” together and “alginate-based” and “medicine” in one search yielded 291,449 results (see Figure 8). Comparatively, in the year 2000, the total sum of the scientific articles for both searches was 1786, whereas the results for alginate-based products were significantly lower (36) than the findings for the phrases “alginate” and “medicine” (1750). By the end of 2022, the total findings raised to 34,800, whereas in May 2023, it was 15,350.

**Figure 9:**
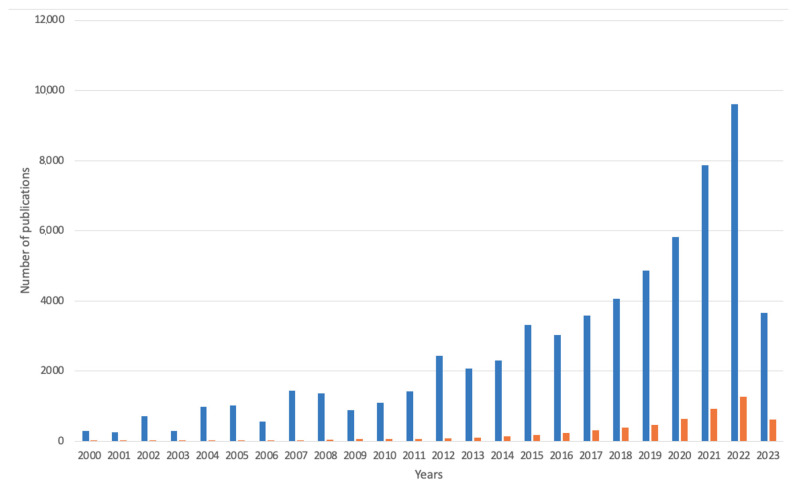
Number of publications over the years on alginate-based materials in cosmetic field (search for words “alginate” and “cosmetics” together) and products based on alginate in this field (search for the phrase “alginate-based” and “cosmetics”). Data are provided from Google Scholar database (in both matters including abstract, title, and keywords); accessed on 18 April 2023.

According to research findings, alginate is commonly used in cosmetics, although in scientific articles, the combination of the words “alginate” and “cosmetics” as well as “alginate-based” with the phrase “cosmetics” yielded fewer results than in medicine and environmental fields—the number of publications in the years 2000–2023 was 68,648 (see Figure 9). The reason for this could be a lack of results published by cosmetic companies. Compared to the year 2000, the list of scientific articles including the mentioned phrases rose from 301 to 10,880 by the end of 2022, whereas in May 2023, the number was 4282.

**Figure 10:**
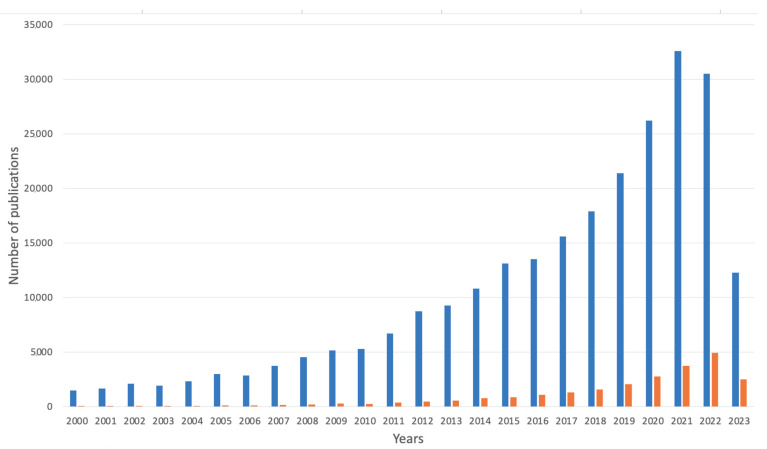
Number of publications over the years on alginate-based materials in food industry field (search for words “alginate” and “food” together) and products based on alginate in this field (search for the phrase “alginate-based” and “food”). Data are provided from Google Scholar database (in both matters including abstract, title, and keywords); accessed on 18 April 2023.

In recent decades, alginate applications in the food industry have gained recognition. In early 2000, the results for “alginate” and “food” as well as alginate-based products in food industry applications described as “alginate-based” and “food” yielded 1505 scientific articles, whereas by the end of 2022, the number was 35,450, and in May 2023, it was 14,780 (see Figure 10).

The comparison of existing knowledge on alginate-based materials indicates that the greatest recognition was in the years 2000–2023, where alginate composites gained applications in environmental remediation (367,047 total results) and the medical field (291,449 total results). The food industry is also evolving, which yielded 277,054 publications on this subject. The lowest number of publications was in the cosmetic field, with 68,648 publications, although the cosmetic industry may not publish many results. The comparison of the number of scientific articles with alginate-based products in the field of environmental protection, medicine, food, and cosmetics in terms of scientific articles showed that the greatest number was published by the environmental (30,767) and medical fields (24,279), whereas fewer publications were available in cosmetic (5692) and food fields (24,334).

## 6. Alginate-Based Materials: The State of Innovation

Current and future applications of alginate are determined by their gel-forming ability in the presence of cations. These undemanding conditions and well-known properties of alginates in both liquid and gel phases make them exceptional compared to other biomaterial polysaccharides. Pectins are supposed to be similar in their sol/gel transition, but this phenomenon is not well-known or commonly used, such as in alginates [174].

Nowadays, sequential structures of alginates and their composition can be manipulated through enzymatic modification. The epimerases used in this process can highly improve the functional properties of alginates as biomaterials and thereby extend their applications [175]. Enzymatically engineered alginates are a promising future issue as they are more elastic, compact, and less permeable, and most importantly, they are eminently stable in physiological conditions. Moreover, the connection between a proper polymer nanostructure and its properties may stand as a model for other polymer-based material systems [175].

Although the alginate hydrogels obtained by non-conventional methods such as cryogelation, non-solvent phase separation, carbon-dioxide-induced and photo-induced gelation, as well as methods using non-metal crosslinkers, constitute an attractive and promising alternative to future developments, these methods require more attention and research to better understand the mechanisms and their directions [176].

Current advances in alginate-based materials using both conventional and non-conventional methods are listed in Table 1.

The food industry is searching for intelligent, active packaging to maintain and monitor the freshness of food, especially meat. Recently, the development of cobalt-based metal–organic nanoparticles that exhibit sensitivity to ammonia and have antimicrobial properties in combination with sodium alginate have helped to control the freshness of food. Shrimp spoilage was monitored by the color of alginate-based material turning from pale pink to brownish black [177]. The novel combination of an alginate–carnauba wax film with calcium ascorbate helped preserve fresh-cut apples for a longer period of time compared to a conventional alginate composite. Carnauba wax improved the water resistance of fresh-cut fruits and sodium ascorbate improved the nutritional value of food, worked as an antioxidant, and prevented apples from browning, whereas glycerin as a plasticizer enhanced the flexibility of the film. Though sodium alginate–carnauba wax film with calcium ascorbate indicated good preservative values for fresh-cut apples, the research should be extended to other types of food for practical applications [181].

Alginate-based materials are also used to prevent environmental pollution. A new-generation biosorbent for the utilization of pesticides has been invented. Novel composites using *Cerastodermaedule* shells with copper and alginate were used to facilitate dynamic thiabendazole pesticide adsorption from water. This alginate-based material with copper indicates a high affinity to benzimidazole molecules, which can effectively adsorb pesticides from wastewater [178]. Recently, the development of a novel catalyst made of zero-valent iron (Fe^0^) dispersed and immobilized on alginate beads with CuO-Fe_3_O_4_ proved to be an efficient material for removing oxytetracycline from wastewater, and the optimal conditions for the degradation of antibiotics were studied [185].

Thiametoxam, the second-generation neonicotinoid insecticide, is considered a major threat to ecosystems. The bioremediation of thiametoxam is possible by using a highly thiametoxam-degradating Gram-negative aerobic bacterium called *Chrysebacteriumsp H5*. The development of polyvinyl alcohol with sodium alginate and biochar, which constitutes an adsorbent with rich porosity, large specific surface area, and great microbial immobilization matrix, resulted in the effective bioremediation of thiametoxam from wastewater [184].

The pollution of used engine oil is considered an environmental threat. The biodegradation of used engine oil can be possible according to novel research. Alginate–attapulgite–calcium carbonate constituted a proper matrix for used engine oil sorption with *Ochrobacterium intermedium LMG 3301*, *Ochrobacterium intermedium LMG 330*, and *Bacillus paramycoides MCCC1A04098 (BC)* immobilization [183].

The invention of a sodium alginate/ß-cyclodextrin/graphene oxide nanocomposite adsorbent enabled the removal of methylene blue dyes, rhodamine b, methyl violet dyes, and methyl orange dyes, which constitute organic environmental pollutants. According to the literature, at proper conditions such as dose and pH value, the removal rate of methylene blue dye using this nanocomposite can reach 84.98% [186]. The mechanism of the dye’s adsorption via the sodium alginate/ß-cyclodextrin/graphene oxide nanocomposite is based on non-covalent (π−π interactions) and electrostatic interactions, as well as hydrogen bonding [187]. The high efficiency of the method using nanocomposite, its biodegradability, and low-cost operations are the reasons it provides significant potential in industrial water treatment and environment remediation [186].

Another invention that allowed the adsorption of methylene blue dye from water solutions was a sodium alginate/acrylic acid/titanium dioxide composite hydrogel, which had an adsorption rate in the range of 91.4–99.4%. The negatively charged surface of titanium dioxide repulses carboxylate ions, which yields more free area for cationic dye adsorption [188].

Graphene oxide/sodium alginate/polyacrylamide ternary hydrogel, due to the significant properties of graphene oxide—such as the capability of creating strong non-covalent interactions with organic dyes and large surface area—makes the composite a great adsorbent in water treatment applications and can be used for environmental protection [189].

Heavy metal water pollution is extremely dangerous for marine ecosystems due to toxicity and bioaccumulation in marine organisms. Heavy metals can pass to water reservoirs from industrial waste and soils as an effect of acidic rain and release heavy metal ions to lakes, rivers, and groundwater, causing major threats to humans and marine life [190].

The adsorption of heavy metal ions represents an open issue in the field of alginate-based materials. Novel alginate–carboxymethyl cellulose gel beads can remove Pb^2+^ ions from wastewater with a 99% adsorption rate. The reason for the high efficiency of alginate–carboxymethyl cellulose gel beads in the removal of Pb (II) is three adsorption mechanisms: chemical, physical, and electrostatic. The chemical mechanism is based on the reaction between functional groups in alginate–carboxymethyl cellulose gel, such as hydroxyl or carboxyl with Pb^2+^, whereas physical adsorption is able to occur because of sodium alginate crosslinking by carboxymethyl cellulose, which provides more adsorption sites. Electrostatic adsorption uses negatively charged gel beads for positively charged Pb^2+^ adsorption on its surface [191]. Ca-alginate entrapped ball-milled biochar showed excellent Cd^2+^-adsorption capacity due to the synergetic effect of calcium alginate and ball-milled biochar [192]. The development of alginate–g-poly(*N*-isopropylacrylamide) graft copolymer allows the removal of Cu (II) from water solutions due to the reaction between carboxyl groups and Cu^2+^ [193]. The plant-mediated biosynthesis of the iron nanoparticles–calcium alginate hydrogel membrane played a significant role in the remediation of Cr (VI), whereas the removal rate of Cr (VI) was 99.5% [194]. Porous alginate beads created by template-assisted emulsion polymerization are efficient for selective Hg^2+^ adsorption, which provides other excellent proof that alginate-based materials play an eminent role in environmental protection [195].

Water pollution by Gram-negative and Gram-positive bacteria and antibiotics is an emerging issue. Using highly adsorptive nanomaterials such as nanosilica or sodium alginate nanoadsorbent, water remediation is possible. Alginate-based materials can remediate antibiotics from groundwater, which could be protective for the environment. The synthesis of calcium/iron-layered double hydroxides–sodium alginate nanoadsorbent as a reactive barrier for antibiotic amoxicillin could be useful in permeable technology adsorption and suitable for removing antibiotics waste from water [196]. Nanosilica adsorbent conduct protein lysozyme adsorption via electrostatic and non-electrostatic interactions, removing both Gram-negative bacteria such as *Escherichia coli*, Gram-positive bacteria such as *Bacillus*, and the antibiotic levofloxacin [197]. Lysozyme is a protein with hydrolytic enzyme properties. It can lyse Gram-positive bacteria, whereas Gram-negative bacteria are protected by an outer membrane; therefore, lysozyme is not able to lyse these kinds of bacteria in vitro without special conditions and factors, such as a proper pH value or the addition of EDTA. Although lysozyme does not have Gram-negative bactericidal properties, it can affect the cytoplasm in *Escherichia coli* and lead to its disintegration [198].

Alginate’s application in medicine abounds with inventions for wound dressings and tissue engineering for nerve regeneration and transplantable skin substitutes. Recently, an alginate hydrogel hemp nonwoven composite was developed for wound healing. Hemp presents good anti-inflammation properties and also constitutes an eco-friendly material [179]. Novel hydrogel based on carboxymethyl chitosan/sodium alginate with simvastatin release ability can be used in the wound healing process. A nanostructure lipid carrier with encapsulated simvastatin with the matrix containing carboxymethyl chitosan and sodium alginate works as a barrier against pathogens, helps to prevent excess effusions, and accelerates the regeneration process. The composite exhibited efficient antibacterial activity against *Staphylococcus aureus* and *Escherichia coli* and indicated biocompatibility on mouse fibroblasts. The results of the study proved that the drug can be released over a prolonged period of time, which can be a major advantage in the wound healing process [199]. As recent studies have shown, an electrospun polycaprolactone/calcium alginate scaffold can be useful in skin tissue engineering. The incorporation of calcium alginate enhances hydrophilicity, fiber crosslinking, and capability to induce attachment for fibroblasts and keratinocyte cells. The non-toxic character of the polymer makes it a suitable material for transplantable skin substitutes [180]. The new technology of carboxyl-modified multi-walled carbon nanotubes was combined with sodium alginate/gelatin composites in order to enhance the scaffold properties in nerve regeneration. The modifications also improved the hydrophilic and mechanical properties of the material as well as electrical conductivity [182].

The potential application of an alginate-based composite gel scaffold doubly integrated with hydroxyapatite and gelatin microspheres crosslinked by a calcium cation in bone tissue engineering was confirmed due to its osteoblast activity. The injectable form constitutes a convenient method for gel administration [200].

Another invention based on incorporating fullerenol nanoparticles into alginate hydrogel to receive an injectable cell medium with antioxidant features for cardiovascular tissue engineering and regenerative medicine applications was discovered in 2017. Using a modified alginate composite with proper mechanical strength, it is possible to reduce reactive oxygen species (ROS) levels and enhance the retention and survivability of implanted brown adipose-derived stem cells, which, through the elicitation of angiogenesis, leads to cardiac function recovery [201]. Robust and double-layer micro-patterned bioadhesive silk nanofibril incorporated with gelatin methacrylate, which promotes the adhesion of corneal stroma cells, could be important in the stroma tissue engineering of the human cornea [202]. Great potential in wound healing could be obtained by scaffolds based on alginate–polyethylene glycol methyl ether methacrylate in combination with natural *Moringa oleifera* and *Aloe vera* aqueous leaf extracts. *Aloe vera*’s ability for increased water uptake, as well as *Moringa oleifera*’s antioxidant capacity and anti-inflammatory properties, and *Streptococcus aureus*’s antimicrobial activity enhances scaffold cell proliferation [203]. Self-crosslinkable oxidized alginate–carboxymethyl chitosan hydrogels could be injectable cell carriers for dental enamel regeneration. Hydrogels were obtained by crosslinking reactions between oxidized alginate aldehyde groups and carboxymehyl chitosan amino groups. The hydrogels exhibited cariogenic antibacterial activity, accelerated the healing process, decreased the risk of infection, and the high in vitro viability of dental epithelial stem cells, which makes them an excellent carrier for cells with growth factors [204]. Alginate-based materials exhibit great potential in oncology; for example, doxorubicin, a model anticancer medication, was successfully encapsulated in alginate–g-poly(*N*-Isopropylacrylamide) micelles and maintained the sustained release of the drug in 37 °C in vitro, reflecting natural human organism conditions [205].

The application of alginate and its modification may even be unexpected because the development of new procedures in the future may generate the need for novel material formation.

## 7. Conclusions

To summarize, alginates are widely used as biomaterials in the medicine, cosmetic and food industries, as well as dentistry. The structure of alginates and, therefore, their properties, can be successfully modified to obtain the desired functions of alginate-based materials.

The alginate-based materials’ capability to incorporate cells and drugs, as well as their promotion of wound healing, highlight them as a promising issue for tissue engineering. Alginate composites, such as alginate–chitosan [206] or alginate–polyethylene glycol [207], alginate–biosilica [208], alginate–ceramics [209], and alginate–proteins, such as collagen, are known for multiply tissue engineering and medicine applications due to the enhanced cell adhesion, biocompatibility, tensile strength, and porosity [210]. Biocompatibility and non-toxicity show that alginate can be safely used in the food and cosmetic industries.

However, there are features that should be improved, for example, obtaining the desired parameters, such as bioactivity, degradation, and mechanical properties simultaneously.

There is an industry demand for emerging alginate-based materials that imitate the environment of natural tissues [176], so the application of alginate in biomedical fields will increase.

Materials that are biodegradable will receive increasing attention in the future, so alginate, which is biodegradable, is an excellent material in this regard.

The comparison of the number of scientific articles with alginate-based products in the field of environmental protection, medicine, food, and cosmetics in scientific articles showed that the greatest number was assigned to the environmental field (30,767) and medicine (24,279), whereas fewer publications were available in cosmetics (5692) and food fields (24,334).

The water pollution issue is highlighted as it constitutes an emerging issue, and thus, seeking new developments based on safe alginate composites that remediate toxic dyes, antibiotics such as amoxycillin, Gram-positive and Gram-negative bacteria, as well as heavy metal ions which can accumulate in marine and human organisms, makes alginate-based materials worthy of attention.

Understanding the chemical structure and physicochemical properties of alginates and their modifications provides new perspectives for many industry branches, such as pharmaceutical, medical, cosmetic, and food industries, as well as dentistry and environmental protection. For decades, researchers have looked for a material that can fulfill their research goals regarding pollution limitation of the natural environment. Due to their biocompatibility, biodegradability, and chelating ability, alginates are currently at the center of such attention.

## Figures and Tables

**Table 1:** Recent advances in alginate-based materials.

Year	Invention	Description	Application	Field	References
2023	SA/Co-MOF	Cobalt-based metal-organic framework (Co-MOF) nanoparticles with ammonia-sensitive and antibacterial functions introduced into sodium alginate (SA) matrix	Intelligent active packaging material	Food industry	[177]
2023	Ce–Cu@Alg	A novel hydrogel-beads-based copper-doped *Cerastodermaedule* shells@Alginate biocomposite for highly fungicide sorption from aqueous medium	New generation biosorbent for utilizing pesticides from wastewater	Environmental protection	[178]
2023	Alginate hydrogel hemp nonwoven composite	An eco-friendly material with anti-inflammatory hemp and alginate hydrogel	Wound dressings	Medicine	[179]
2023	(PCL)/calcium alginate (CA)	Three-dimensional fibrous scaffolds consisting of poly(ε-caprolactone) and calcium alginate used to induce keratinocyte differentiation through the action of calcium leaching	Transplantable skin substitutes	Tissue engineering	[180]
2023	Sodium alginate–carnauba wax film containing calcium ascorbate	A new type of edible composite film with water-blocking agent carnauba wax, plasticizer glycerin, antioxidant, and nutritional enhancer sodium ascorbate on the basis of traditional sodium alginate composite film	Fresh-cut fruit preservation	Food industry	[181]
2023	Alg/Gel/mMWCNTs conductive scaffolds	Novel hybrid conductive scaffold based on alginate/gelatin/carboxylated carbon nanotubes	Nerve regeneration	Tissue engineering	[182]
2023	(AAC) gel	Alginate–attapulgite–calcium carbonate gel adsorption in bacterial biodegradation of used engine oil	Adsorptive granular formulas for bioremediation of used engine oil	Environmental protection	[183]
2023	PVA/SA/biochar beads with *Chryseobacterium* sp H5 immobilization	Polyvinyl alcohol (PVA)/sodium alginate (SA)/biochar bead with functional microbe immobilization	Effective bioremediation of thiamethoxam contamination	Environmental protection	[184]
2023	(CuO-Fe_3_O_4_-Fe^0^/Abs)	Fe^0^ Embedded alginate beads and coated with CuO-Fe_3_O_4_	Sustainable catalyst for photo-Fenton degradation of oxytetracycline in wastewater	Environmental protection	[185]
2017	SCGG	Sodium alginate/ß-cyclodextrin/graphene oxide nanocomposite adsorbent	Bioremediation of dyes	Environmental protection	[186,187]
2016	SA/acrylic acid/TiO_2_	Sodium alginate/acrylic acid/titanium dioxide composite hydrogel	Bioremediation of dyes	Environmental protection	[188]
2013	GO/SA/polyacrylamide ternary hydrogel	Graphene oxide/sodium alginate/polyacrylamide ternary hydrogel	Bioremediation of dyes	Environmental protection	[189,190]
2016	SA-CMC	Alginate–carboxymethyl cellulose gel beads	Bioremediation of heavy metals: Pb (II)	Environmental protection	[191]
2018	CA-BMB	Ca-alginate entrapped ball-milled biochar	Bioremediation of heavy metals Cd^2+^	Environmental protection	[192]
2019	Alg-g-PNIPAAm	Alginate-g-poly(*N*-isopropylacrylamide) graft copolymer	Bioremediation of heavy metals Cu (II)	Environmental protection	[193]
2019	FeNPs-CaAlg	Iron nanoparticles–calcium alginate hydrogel membrane	Bioremediation of heavy metals: Cr (VI)	Environmental protection	[194]
2019	Alg-B	Porous alginate beads	Bioremediation of heavy metals: Hg^2+^	Environmental protection	[195]
2023	(Ca/Fe)-LDH-SA beads	Calcium/iron-layered double hydroxides–sodium alginate nanoadsorbent	Bioremediation of antibiotics: Amoxicillin	Environmental protection	[196]
2023	Lys-protein nanomaterial	Lysozyme protein modified nanomaterials	Bioremediation of bacteria and antibiotics	Environmental protection	[197,198]
2023	Carboxymethyl chitosan/sodium alginate hydrogel	Hydrogel based on carboxymethyl chitosan/sodium alginate with the ability to release simvastatin for chronic wound healing	Wound healing	Medicine	[199]
2016	Hap-GMs-CaCO_3_-GDL	Injectable alginate/hydroxyapatite gel scaffold combined with gelatin microspheres	Bone tissue regeneration	Tissue engineering/ Medicine	[200]
2017	Fullerenol/Alg Hydrogel	Injectable fullerenol/alginate hydrogel	Cardiovascular tissue regeneration	Tissue engineering/ Medicine	[201]
2021	Silk nanofibril/GelMA-alginate	Double-layer micro-patterned bioadhesive based on silk nanofibril/GelMA–alginate	Stroma tissue engineering of human cornea	Tissue engineering/ Medicine	[202]
2019	Ca-alginate-PEGMA/*A. vera*/*M. oleifera*	Alginate–PEG methyl ether methacrylate–moringa oleifera–aloe vera scaffolds	Wound healing	Tissue engineering/ Medicine	[203]
2022	OA-CC hydrogels	Self-crosslinkable oxidized alginate–carboxymethyl chitosan hydrogels	Dental enamel regeneration	Tissue engineering/ Dentistry	[204]
2014	PNIPAAm	Doxorubicin-loaded alginate-g-poly(*N*-Isopropylacrylamide) micelles	Cancer imaging and therapy	Medicine	[205]

## Data Availability

No new data were created or analyzed in this study. Data sharing is not applicable to this article.
